# Clinical evaluation of the Immulite® 1000 chemiluminescent immunoassay for measurement of equine serum insulin

**DOI:** 10.3389/fvets.2023.1018230

**Published:** 2023-03-27

**Authors:** Yun Young Go, Nicole W. Hazard, Udeni B. R. Balasuriya, Anna M. Chapman, Nanette S. Fitton, Ákos Kenéz, Frank M. Andrews

**Affiliations:** ^1^Department of Infectious Diseases and Public Health, Jockey Club College of Veterinary Medicine and Life Sciences, City University of Hong Kong, Kowloon, Hong Kong SAR, China; ^2^Louisiana Animal Disease Diagnostic Laboratory and Department of Pathobiological Sciences, School of Veterinary Medicine, Louisiana State University, Baton Rouge, LA, United States; ^3^Equine Health Studies Program, Department of Veterinary Clinical Sciences, School of Veterinary Medicine, Louisiana State University, Baton Rouge, LA, United States; ^4^Siemens Healthcare Diagnostics Inc., Tarrytown, NY, United States

**Keywords:** insulin dysregulation, equine metabolic syndrome, quantitative measurement, validation, equine serum insulin, horse

## Abstract

**Introduction:**

Accurate quantitative analysis of equine insulin in blood samples is critical for assessing hyperinsulinemia in horses. Although there are various laboratory methods for evaluating equine serum insulin, different immunoassays show significant discrepancies between the determined insulin concentrations and are often not comparable. The aim of this study was to evaluate the Immulite® 1000 chemiluminescent immunoassay (CLIA) to establish independent laboratory and assay-specific cut values to provide an accurate diagnosis of hyperinsulinemia in horses. Thus, the analytical and clinical performance of Immulite® 1000 CLIA in terms of precision (intra- and inter-assay coefficient of variance, CV) and recovery upon dilution were evaluated and compared with radioimmunoassay (RIA), which has been previously validated for use in horses.

**Material and methods:**

Archived serum samples (*n* = 106) from six Quarter horse mares enrolled in the glucose phase of a Frequently Sampled Insulin and Glucose Test (FSIGT) study were used to measure blood insulin.

**Results:**

The Immulite® 1000 CLIA had good precision with acceptable intra- and inter-assay CVs, adequate recovery on dilution, and a strong correlation with the RIA (*r* = 0.974, *P* < 0.0001), with constant bias resulting in consistently lower values.

**Discussion:**

On this basis, the Immulite® 1000 Insulin Assay is valid for measuring equine serum insulin for diagnostic and monitoring purposes when cut values are appropriately adjusted.

## 1. Introduction

Equine metabolic syndrome (EMS) has increasingly become a common problem in modern equine clinical practice (1–4). EMS is a complex disorder recognized in horses and ponies, which includes increased regional or general adiposity, insulin dysregulation, and predisposition to laminitis (5–8). Laboratory diagnosis of EMS is based on the quantitative analysis of insulin levels in blood samples, which is critical to assessing dysregulation of glucose and insulin homeostasis in horses (9, 10). Most horses with insulin dysregulation have tissue insulin resistance with compensatory hyperinsulinemia and a broad range of serum insulin concentrations (11–14). Thus, an ideal diagnostic test should be able to determine accurate results encompassing a broad range of values. However, the lack of equine-specific standards or access to a gold-standard assay makes assessing accurate EMS-related changes in insulin regulation difficult. Various laboratory methods are employed for quantitative analysis of insulin in equine serum or plasma, including enzyme-linked immunosorbent assay (ELISA), radioimmunoassay (RIA), and chemiluminescent immunoassay (CLIA). Among these assays, human-specific insulin RIA validated for use in horses was the most commonly used in clinical practice and experimental studies. Consequently, the reference intervals currently used for the diagnosis of insulin dysregulation were established from studies using RIA; however, these assays have limited commercial availability. More importantly, studies indicated that different immunoassays are not comparable and showed significant differences between the determined insulin concentrations, making result interpretation and EMS-related insulin diagnosis difficult (15, 16). Therefore, establishing an independent laboratory and assay-specific reference intervals or cutoff values is critical to providing an accurate diagnosis and for the monitoring of insulin dysregulation in EMS patients (17).

The CLIA is a convenient and commonly used method for quantitative analysis of insulin in commercial laboratories; however, comprehensive validation in horses has not been done. In this study, the validation of the Immulite® 1000 Insulin Assay (Siemens, USA) to measure insulin concentrations in equine serum samples was performed thoroughly. To do this, we evaluated the analytical and clinical performance of Immulite® 1000 CLIA in terms of precision (intra- and inter-assay coefficient of variance, CV), recovery upon dilution, and its comparison with RIA that was previously validated for measurement of insulin concentration in horses.

## 2. Materials and methods

### 2.1. Animals and blood samples

Archived serum samples from six Quarter horse mares from the Louisiana State University AgCenter Teaching and Research Herd, previously diagnosed with EMS, were used to measure serum insulin. Samples were collected from horses enrolled in a Frequently Sampled Insulin and Glucose Test (FSIGT) study conducted in June 2019. Briefly, horses were administered dextrose solution (50%, IV; 300 mg/kg body weight) in that study. After 20 min of dextrose administration, horses were given insulin (Novolin® 0.015 IU/kg body weight, IV). Jugular blood samples used for validation were collected at 0, 1, 2, 3, 4, 5, 6, 7, 8, 9, 10, 11, 12, 13, 14, 16, 18, and 20 min before and during dextrose administration and prior to insulin administration. Samples were allowed to clot at room temperature, and collected serum was stored at −80°C for 11 months until assayed (18).

### 2.2. Immunoassays (CLIA and RIA)

A human insulin-specific radioimmunoassay (Human Insulin Specific RIA kit, Millipore Corporation), previously validated for use with horse serum, was used to measure insulin concentrations as described previously (19). The RIA was performed with reagent volumes and incubation times described in the manufacturer's protocol. The low insulin (mean 10.6 ± 1.78 μIU/ml, CV 16.8%) and high insulin (mean 56.1 ± 5.36 μIU/ml, CV 9.4%) standards were run at the beginning and end of each assay.

For the preparation of insulin standards, the Immulite® Systems Insulin Calibration Verification Material (CVM; Siemens, Germany), traceable to the World Health Organization (WHO) NIBSC 1st International Reference Preparation (IRP) 66/304, was used following the manufacturer's instructions. Briefly, four levels of lyophilized insulin standards in an equine serum-based matrix were reconstituted in deionized water to reach expected target values as indicated by the manufacturer's instructions. Quantitative analysis of insulin was performed on the Immulite® 1000 insulin solid-phase chemiluminescent assay, Immulite® 1000 system (Siemens Healthineers, Germany), following the manufacturer's instructions.

### 2.3. Evaluation of Immulite® 1000 CLIA (linearity, precision, and recovery)

The linearity of the assay was assessed using equine serum-based insulin control solutions at four expected concentrations (0, 5.4, 24.8, and 309 μIU/ml). The intra-assay CV of the immunoassay was evaluated with 10 replicates of two nonhuman serum-based insulin control solutions, one at low (target mean 9.2 μIU/ml) and one at medium (target mean 47 μIU/ml) concentrations. The two insulin control solutions were also assayed in duplicates on 34 different assay runs to calculate the inter-assay CV. The precision (μIU/ml) for the manufacturers of the Immulite® 1000 showing acceptable coefficient of variation percent (CV%) of <10% at each measurement level within run and total (Table 1). Insulin concentrations for each solution were measured, then means and standard deviations of the replicate values were calculated. In addition, precision was determined using three insulin concentrations [low, medium, and a mix of the two (target mean 28.1 μIU/ml)] prepared in 10 aliquots initially, then each was thawed once and used as the same samples over five runs.

**Table 1:** Precision (μIU/ml) for the manufacturers of the Immulite® 1000 showing acceptable coefficient of variation percent (CV%) of <10% at each measurement level within run and total.

**Within run**				**Total**	
**Level**	**Mean (**μ**IU/ml)**	**SD**	**CV (%)**	**SD**	**CV (%)**
1	7.39	0.47	6.4	0.59	8.0
2	12.30	0.65	5.3	0.74	6.0
3	17.80	1.08	6.1	1.26	7.1
4	25.50	1.46	5.7	1.50	5.9
5	102.00	5.26	5.2	6.20	6.1
6	300.00	15.8	5.3	21.0	7.0

The recovery upon dilution (RUD) and the linearity of dilution were calculated to evaluate accuracy. A high concentration of insulin control solution (309 μIU/ml) was serially diluted two-fold from neat to 1:256 with Immulite® 1000 insulin diluent buffer to achieve the expected concentration range of 1.2–309 μIU/ml. Observed total error (TE_O_; %) was obtained by |bias (%)| + 2 CV (%) as described elsewhere (20). Samples were run in triplicate. Percentage recovery was calculated as the observed concentration/expected concentration × 100.

### 2.4. Comparison of Immulite® 1000 CLIA to RIA

A total of 106 equine serum samples representing a wide range of insulin concentrations were assayed simultaneously by the Immulite® 1000 CLIA and the RIA. The maximum reportable concentrations for the Immulite® 1000 CLIA and RIA were 300 and 200 μIU/ml, respectively; thus, any samples with insulin concentration >200 μIU/ml with RIA were excluded from statistical analysis.

### 2.5. Statistical analysis

Data analysis was performed using GraphPad Prism software v.9.2.0. Linearity was assessed by linear regression to determine the slope and intercept of the line in comparison with 1 and 0, respectively. The coefficient of variation (%) was calculated as the ratio of the standard deviation to the mean multiplied by 100. A CV ≤ 10% was considered acceptable (21). The least-square linear regression was performed to evaluate the relationship between the RIA and CLIA. A Pearson correlation coefficient was calculated for the 106 samples analyzed by both methods. Bland–Altman analysis was performed to calculate method-dependent bias and limits of agreement between Immulite® 1000 CLIA and RIA (22). Sensitivity and specificity of the CLIA-based values were calculated with reference to the RIA-based diagnostic outcome. Sensitivity was calculated as [number of true positives/(number of true positives + number of false negatives)] × 100 and specificity was calculated as [number of true negatives/(number of true negatives + number of false positives)] × 100. The positive predictive value (PPV) was calculated using the equation: [number of true positives/(number of true positives + number of false positives)] × 100 and negative and the negative predictive value (NPV) was calculated as follows: [number of true negatives/(number of false negatives + number of true negatives)] × 100 (23).

## 3. Results

### 3.1. Validation of Immulite® 1000 CLIA

**Figure 1:**
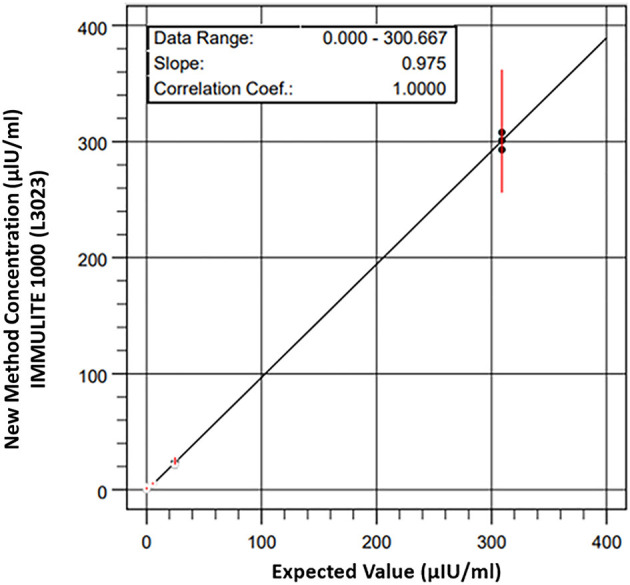
Linearity of standard reference reagents for equine serum based-insulin solutions measured by Immulite® 1000 CLIA using equine serum-based control solutions was excellent, showing a strong relationship between the expected and measured concentrations of the diluted samples. Linear regression at analyzed concentrations indicated the best fit line as *y* = 0.975 *x* – 0.585 (*r* = 0.999, *P* < 0.0001). The analytical range of the Immulite® 1000 CLIA was 0 to 300 μIU/ml.

The linearity using equine serum-based control solutions was excellent, showing a strong relationship between the expected and measured concentrations of the diluted samples. Linear regression at analyzed concentrations indicated the best fit line as *y* = 0.975 *x* – 0.585 (*r* = 0.999, *P* < 0.0001). The analytical range of the Immulite® 1000 CLIA was 0–300 μIU/ml (Figure 1). The intra-assay CV was within acceptable limits, with 4.6% for samples at low mean insulin concentrations of 8.2 ± 0.37 μIU/ml and 5.5% for samples at medium serum-based insulin concentrations of 42.2 ± 2.34 μIU/ml. The inter-assay CV from two control samples were 7.4% for the low concentration control (mean 9.1 ± 0.68 μIU/ml) and 7.7% for the medium concentration control (mean 47.7 ± 3.69 μIU/ml). The mean % CV for three Immulite® 1000 control samples subjected to a freeze-thaw cycle was 6.9% for the low control (mean 8.0 ± 0.56 μIU/ml), 3.3% for the medium control (mean 41.2 ± 1.38 μIU/ml), and 1.8% for the mixture of low and medium controls (mean 25.6 ± 0.46 μIU/ml).

**Figure 2:**
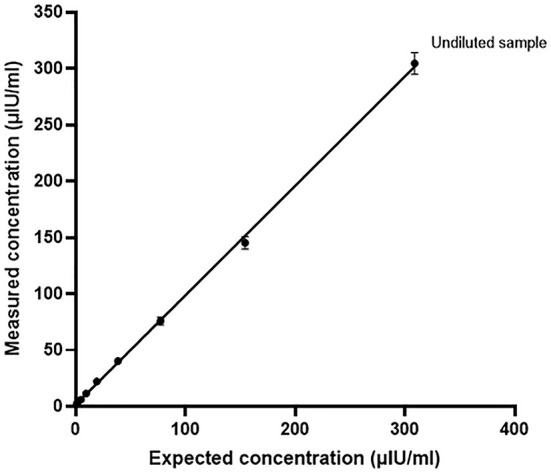
Recovery upon dilution (RUD) of nonhuman serum-based insulin control solutions with Immulite® 1000 CLIA. The RUD was calculated as a percentage of recovery of the insulin concentration in the two-fold serially diluted samples with the corresponding expected concentrations. Samples were diluted with commercially available insulin diluent buffer.

Lastly, the mean percentage recovery from the initial high concentration (309 μIU/ml) of nonhuman serum-based insulin control diluted with commercially available sample buffer was calculated at different dilution factors (Table 2). The percentage recovery increased as the dilution factor increased, with the corresponding range of recovery stretching from 94.1 to 206.8%. When samples were subdivided into concentration range groups of high-medium (38.6–309 μIU/ml) and low (1.2–19.3 μIU/ml) concentrations, the overall mean percentage recovery was 98.6% ± 3.5 and 147.4% ± 37.7, respectively. The linearity upon dilution was excellent (*r*^2^ = 0.9981; *p* < 0.0001), showing a strong relationship between the calculated and measured concentrations of the diluted samples (Figure 2).

**Table 2:** Concentrations of serially diluted insulin (μIU/ml) determined by Immulite® 1000 CLIA showing dilution, replicate, observed concentration, mean, expected concentration, total observed error percent (TEo%, from Table 4) and observed/expected percent.

**Dilution**	**Replicate**	**Observed concentration (μIU/ml)**	**Mean**	**Expected concentration (μIU/ml)**	**TE_O_ (%)**	**Observed/expected %**
1:1	1	315	304.7	309		98.6
	2	303			7.7	
	3	296				
1:2	1	139	145.3	154.5		94.1
	2	150			13.8	
	3	147				
1:4	1	77.4	75.7	77.3		98.0
	2	71.6			11.6	
	3	78.2				
1:8	1	40.1	40.1	38.6		103.9
	2	38.2			13.6	
	3	42.1				
1:16	1	23.7	22.1	19.3		114.3
	2	20.5			29.0	
	3	22				
1:32	1	11	11.4	9.7		118.4
	2	12.2			29.2	
	3	11.1				
1:64	1	5.49	5.8	4.8		120.2
	2	5.29			45.7	
	3	6.63				
1:128	1	3.41	4.3	2.4		177.3
	2	3.61			141.7	
	3	5.82				
1:256	1	2.96	2.5	1.2		206.8
	2	2.21			140.8	
	3	2.32				

The observed/expected percent was acceptable when it was between 80 and 120%. Insulin values at the low range <20 μIU/ml were considered too variable and dilution at 1:32 was not acceptable.

### 3.2. Comparison between Immulite® 1000 CLIA and RIA

**Figure 3:**
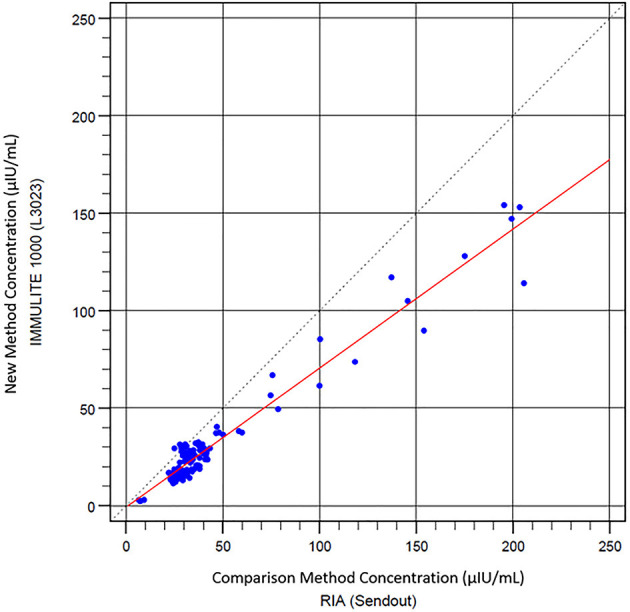
Scatter plot of Immulite® 1000 CLIA compared with RIA results. Least square regression analysis indicates a best-fit line (red) *y* = 0.713 *x* – 0.721.

**Figure 4:**
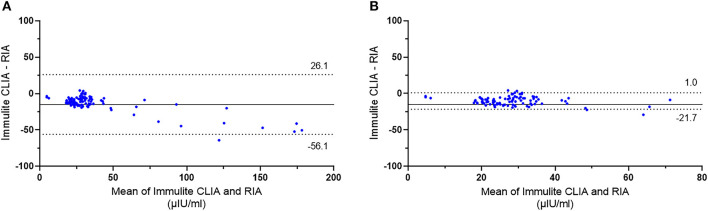
Bland–Altman plot showing agreement between insulin concentrations (μIU/ml) in equine serum samples measured with Immulite® 1000 CLIA and RIA. **(A)** All samples (*n* = 106) and **(B)** samples with RIA <100 μIU/ml (*n* = 95). The solid line indicates the bias, and the dotted lines indicate the 95% limits of agreement.

Next, the equine serum insulin concentrations measured with Immulite® 1000 CLIA were compared to those of the RIA. Scatter plots of CLIA concentrations against RIA concentrations with a line of best fit derived by the least-squares regression analysis are shown in Figure 3. There was a strong positive correlation between Immulite® 1000 CLIA and RIA (*r* = 0.974, *P* < 0.0001). The gradient (95% CI) of the best fit line was 0.713 (CI, 0.68–0.75), and the intercept was −0.721. The results from the regression analysis and the difference plots indicated that the CLIA results were in good agreement with RIA. A Bland–Altman plot of all samples showed a constant bias with Immulite® 1000 concentrations, a mean of −15.01 ± 20.99 μIU/ml lower than RIA concentrations, and with 95% limits of agreement (LOA) of −56.1 to 26.1 μIU/ml (Figure 4A). A small proportional error was detected with the difference plot between the CLIA and RIA samples with high insulin concentrations. When only samples with RIA <100 μIU/ml were analyzed, a constant bias was present with Immulite® 1000 concentrations, a mean of −10.3 ± 5.79 μIU/ml lower than RIA concentrations, with 95% LOA of −21.6 to 1.0 μIU/ml (Figure 4B). The sensitivity and specificity of the Immulite® 1000 CLIA insulin concentrations were higher when the diagnostic cutoff was lowered from 20 to 10 μIU/ml (Table 3).

**Table 3:** Sensitivity, specificity, and positive and negative predictive values for Immulite® 1000 CLIA insulin concentrations compared to RIA using two diagnostic cutoffs.

**Diagnostic cutoff**	**Number of samples**	**Sensitivity (%)**	**Specificity (%)**	**Positive predictive value (%)**	**Negative predictive value (%)**
>10 μIU/ml	106	100	100	100	100
>20 μIU/ml	106	61	100	100	7

## 4. Discussion

In this study, analytical and clinical evaluations of the Immulite® 1000 CLIA had acceptable precision based on manufacturers claims, both within and between runs and at a wide range of clinically relevant insulin concentrations. The intra-assay and inter-assay CVs of Immulite® 1000 CLIA determined in our study were comparable to the reported precision of other immunoassays, including those of RIAs that ranged from 4.4 to 10.7% (15). In addition, CLIA offered an analytical range of 2–300 μIU/ml that allowed coverage of a broader range of insulin concentrations compared to the RIA (0–200 μIU/ml). The RUD results showed that the Immulite® 1000 CLIA allows valid measurements of samples with a mean recovery rate of 101.8% ± 6.9 within a range of 19.3–309 μIU/ml insulin concentrations. However, the mean recovery rate was too high in the low concentration (1.2–19.3 μIU/ml) samples, as the assay consistently overestimated the insulin values in samples <20 μIU/ml. Furthermore, CLIA accuracy was assessed by comparison with the RIA. The RIA has been the most commonly used method in experimental studies and has been shown to be the most appropriate among other immunoassays for routine evaluation (15). As a result, serum insulin concentrations measured with the Immulite® 1000 CLIA showed a strong and positive correlation in comparison with the RIA that has been previously validated for use in horses. When method-dependent bias and limits of agreement were assessed, there were small constant biases, where Immulite® 1000 CLIA measured insulin concentrations were generally underestimated compared to those of the RIA. Calculating the biases at the different levels in the recovery study Table 4, at expected 9.7 have positive bias of 17.5%, at 19.3, positive bias of 14.5%, at 38.6, positive bias of 8.9%, at 77.3, negative bias of 2.1%, at 154.5, negative bias of 6.0% and at 309, negative bias of 1.4%. This matches up with the Bland–Altman plot suggesting that results of ~40 μIU/ml have little bias. There was a positive bias at the lower levels of 14.5 to 17.5% (Table 4). Using the regression equation and 20 μIU/ml cut off threshold, the bias was 32%, at 65 μIU/ml threshold, bias was 30% and at 100 μIU/ml threshold, bias is 30%. However, the variations were with acceptable range when values were >40 μIU/ml.

**Table 4:** Insulin values (μIU/ml) in triplicate at each level showing individual values, mean values, range, standard deviation (SD), CV% (coefficient of variation percent), percent bias (bias%), and total observed error (TEo%).

**Observed concentrations and range (μIU/ml)**	**Mean (μIU/ml)**	**Expected concentration (μIU/ml)**	**Mean (μIU/ml)**	**SD (%)**	**CV (%)**	**Bias (%)**	**TEo (%)**
315	304.7	309	304.7	9.6	3.15%	1.39%	7.7%
303							
296							
139	145.3	154.5	145.3	5.7	3.91%	5.95%	13.8%
150							
147							
77.4	75.7	77.3	75.7	3.6	4.76%	2.07%	11.6%
71.6							
78.2							
40.1	40.1	38.6	40.1	2.0	4.86%	3.89%	13.6%
38.2							
42.1							
23.7	22.1	19.3	22.1	1.6	7.26%	14.51%	29.0%
20.5							
22							
11	11.4	9.7	11.4	0.7	5.82%	17.53%	29.2%
12.2							
11.1							
5.49	5.8	4.8	5.8	0.7	12.46%	20.83%	45.7%
5.29							
6.63							
3.41	4.3	2.4	4.3	1.3	31.25%	79.17%	141.7%
3.61							
5.82							
2.96	2.5	1.2	2.5	0.4	16.22%	108.33%	140.8%
2.21							
2.32							

TEo% values of <25% were considered acceptable at insulin values of 40 μIU/ml and higher. TEo% values for insulin were >25% in insulin samples <40 μIU/ml. Observed total error (TEO; %) was calculated as |bias (%)| + 2 CV.

Thus, to be confident based on the constant bias and TEo at the various cutoff points described in the consensus statement, at the 20 μIU/ml cutoff, a result above 26 μIU/ml, at the 40 μIU/ml, a result above 48 μIU/ml, should be used. The slight differences observed from lower concentration samples between the two immunoassays were also reflected in the gradients and intercepts seen from the regression analysis line of best fit. In contrast, the range of variation decreased significantly when only samples with insulin concentrations at the range most commonly used for diagnostic purposes (0–100 μIU/ml) were included in the analysis. The total error associated with the various cutoffs recommended by the equine endocrine group and the measurement uncertainty (2 × CV) was acceptable. At the cutoff of 20 μIU/ml, the Total observed error (TEo) was 29%, at the cutoff of 40 μIU/ml was 13.6%, and at the cutoff 75 μIU/ml the TEo was 11.6%. Therefore, there was agreement in the clinical application of the diagnostic cutoffs purposed for insulin, which would be considered acceptable at an TEo of 20%−30%. However, when samples were in the “grey zone” of TEo was close to the cutoff value of 30%. This was an expected finding as values in horses between 20–50 μIU/ml warrant further dynamic testing to confirm a diagnosis of insulin resistance.

Many of the reference intervals commonly used in clinical practice and experimental studies to diagnose equine hyperinsulinemia are based on studies using the RIA (24–26). In this study, a comparative analysis between Immulite® 1000 CLIA and RIA showed that the CLIA results were consistently lower than the results provided by the RIA. It is also reflected in the lower sensitivity and negative predictive values of the Immulite® 1000 CLIA determined by using the 20 μIU/ml cutoff value (Table 3), which is stated in the consensus statements of the American College of Veterinary Internal Medicine for diagnosis of fasted hyperinsulinemia in horses (1). In contrast, CLIA showed 100% agreement with the RIA results regarding sensitivity and specificity when a 10 μIU/ml cutoff value was used, suggesting that the independent laboratory and assay-specific cutoff values should be applied in agreement with previously reported adjustments of chemiluminescent-based diagnostics (17). The tendency for CLIA to provide lower measurements of insulin concentrations compared to the RIA, in particular for samples with concentrations under 100 μIU/ml, was also described in previous studies (27, 28). Similar to our results, differences in insulin concentrations between the human-specific RIA and the commercially available CLIA, which is often used for the measurement of equine insulin, was previously described by Banse et al. (16). They observed poor agreement between both assays for measuring equine insulin, which could be attributed to decreased antibody binding to equine insulin compared to human insulin (16). There are two amino acid differences in equine insulin molecules compared to human insulin (29, 30). It is plausible that these slight differences in the amino acid sequence result in conformational changes of the antibody binding site causing reduced binding affinity and negatively impacting the sensitivity of insulin immunoassays. Unfortunately, there are no immunoassays currently available that use antibodies that have been specifically raised against equine insulin or equine insulin standards.

In conclusion, the Immulite® 1000 CLIA showed imprecision within manufacturer's claims and that constant negative bias was apparent in the CLIA assay compared to the RIA. There was an increasing negative proportional bias that became most apparent above 75 μIU/mL with the CLIA assay but is unlikely to be of clinical significance since 75 is well above the cutoff of 65 recommended by the Equine Endocrinology Group for dynamic testing. The diagnostic thresholds of 20 and 50 or 75 recommended for identification of patients needing further dynamic testing had some associated variation, but this only was significant if the results were close to the cutoff and “grey zones” associated with total error or analytical variation (measurement uncertainty). Therefore, it might be possible to use the RIA developed cutoffs for CLIA Assay with the Immulite 1000®, however, in contrast, CLIA showed 100% agreement with the RIA results regarding sensitivity and specificity when a 10 μIU/ml cutoff value was used, suggesting that independent laboratory and assay-specific cutoff values should be applied in agreement with previously reported adjustments of chemiluminescent-based diagnostics.

## Data availability statement

The original contributions presented in the study are included in the article/supplementary material, further inquiries can be directed to the corresponding author.

## Ethics statement

The animal study was reviewed and approved by Louisiana State University Institute of Animal Care and Use Committee (LSU IACUC).

## Author contributions

NH, AC, and NF contributed to the study design, execution, and data analysis. FA, UB, YG, and ÁK contributed to the study design, data analysis and interpretation and manuscript preparation. All authors had full access to all data in the study, took responsibility for the integrity of the data and the accuracy of data analysis, and gave their final approval of the manuscript.
